# V-set and immunoglobulin domain containing 1 (VSIG1) as an emerging target for epithelial–mesenchymal transition of gastric cancer

**DOI:** 10.1038/s41598-022-19883-1

**Published:** 2022-09-28

**Authors:** Catalin-Bogdan Satala, Ioan Jung, Zsolt Kovacs, Raluca-Ioana Stefan-Van Staden, Calin Molnar, Tivadar Bara, Andrei-Ionut Patrichi, Simona Gurzu

**Affiliations:** 1 Department of Pathology, George Emil Palade University of Medicine, Pharmacy, Science and Technology, Targu Mures, Romania; 2 Department of Biochemistry, George Emil Palade University of Medicine, Pharmacy, Science and Technology, Targu Mures, Romania; 3 National Institute of Research for Electrochemistry and Condensed Matter, Bucharest, Romania; 4 Department of Surgery, George Emil Palade University of Medicine, Pharmacy, Science and Technology, Targu Mures, Romania; 5 Research Center for Oncopathology and Translational Medicine (CCOMT), George Emil Palade University of Medicine, Pharmacy, Science and Technology, Targu Mures, Romania

**Keywords:** Cancer, Biomarkers

## Abstract

V-set and Immunoglobulin domain containing 1 (VSIG1) is a cell–cell adhesion molecule which role in the genesis and evolution of gastric cancer (GC) is not understood. Only three Medline-indexed papers have focused on the role of VSIG1 in GC. The clinicopathological features of 94 GCs were examined in association with immunohistochemical (IHC) patterns of VSIG1, E-cadherin, and β-catenin which were assessed in the tumor core (central) vs. invasive edge. Cases were classified depending on the VSIG1 expression: membrane/membrane in both core and invasive front; null/negative staining in both core and invasive front; and cases with translocational patterns: membrane core/cytoplasmic buds and cytoplasmic core/null buds. Most of the tumors showed null pattern (n = 54). Cases with translocational patterns (n = 20) were GCs with a high lymph node ratio value (≥ 0.26) and advanced Dukes-MAC-like stage. Of the 20 total cases, 9 showed membrane-to-nuclear translocation of β-catenin and loss of E-cadherin, as indicators of epithelial–mesenchymal transition. All cases with membrane/membrane pattern (n = 20) involved the distal stomach. The poorest overall survival was registered in patients with subcellular translocation of VSIG1, compared to those with either membrane/membrane or null patterns (p = 0.002). In GC, VSIG1 acts as an adhesion membrane protein but its membrane-cytoplasmic translocation can be an indicator of epithelial–mesenchymal transition due to cytoplasmic VSIG1-mediated activation of canonical Wnt/β-catenin signaling pathway.

## Introduction

Despite major improvements in diagnostic and therapeutic tools in oncology, gastric cancer (GC) remains one of the top causes of cancer-related deaths worldwide. More than one million new cases were diagnosed in 2020, and almost 770,000 patients with GC died in that year^1^. GC resistance to chemotherapy is partially explained by its being one of the most heterogeneous tumors from the phenotypic, genetic and molecular points of view^2,3^.

The first classification of GC was established by Lauren more than 50 years ago and is still in use^4^. In recent decades, many molecular classification systems, such as those suggested by The Cancer Genome Atlas (TCGA) and the Asian Cancer Research Group (ACRG) have been proposed^5,6^.

Several biomarkers with possible prognostic or predictive impact in GC have also been identified in the last decade. One of these biomarkers is V-set and Immunoglobulin domain containing 1 (VSIG1), a cell surface protein in the junction adhesion molecule (JAM) family that is encoded by *VSIG1* gene located on chromosome Xq22.3^7^. Also known as glycoprotein A34 (GpA34), VSIG1 was firstly characterised by Scanlan et al.^8^. Although it was initially considered a gastric-restricted biomarker, its presence was further demonstrated in other normal and neoplastic tissues, such as ovary, testis, lung and liver^9–11^.

In gastric mucosa, VSIG1 was shown to be indispensable for normal glandular differentiation, but the relevance of its subcellular localization (membrane vs. cytoplasm) in gastric tumor cells is unknown^10,12^. To the best of our knowledge, between 2006 and 2021, only three studies were focused on the role of VSIG1 in GC. The authors of these studies concluded that VSIG1 is a cell adhesion molecule which loss of expression in tumor cells is associated with a worse prognosis^10,12,13^. The mechanism of its loss has not yet been described.

The purpose of this study was to examine the potential prognostic role of VSIG1 in GC patients, based on its pattern of expression in the tumor core and the invasive edge, which is also known as the tumor invasion front. Whether VSIG1 expression was associated with epithelial–mesenchymal transition (EMT) was also assessed.

## Materials and methods

### Selection of cases

This is an observational study that enrolled 94 randomly selected patients with GC diagnosed between 2017 and 2021 in the Department of Pathology, Clinical Emergency Hospital of Targu Mures, Romania. The present study was approved by the Ethics Committee Board of the Clinical Emergency Hospital of Targu Mures, Romania and was exempted from informed consent for those included retrospectively. All methods were performed in accordance with the relevant guidelines and regulations of our institution.

The inclusion criteria were surgically treated GC cases (subtotal or complete gastrectomy, with D2/D3 lymphadenectomy), with at least 3 months postoperative survival and without adjuvant therapy prior to intervention. Patients with tumors other than intestinal and diffuse type carcinoma (e.g. hepatoid carcinoma, choriocarcinoma, plasmacytoid carcinoma), inoperable or recurrent carcinomas, or metastatic tumors of the stomach were excluded.

### Tissue microarray blocks

For all cases, the histological slides were reviewed, and tumor buds were counted for adenocarcinomas. Then, after microscopic analysis of all tumor sections stained with haematoxylin and eosin (HE), the most representative slide from each case, with at least 80% viable tumor cells and without necrosis or haemorrhage was chosen. For tissue microarray (TMA) blocks, two representative areas were marked for each case: one from the tumor core and one from periphery (invasion front). These two areas of interest were removed from the formalin-fixed paraffin-embedded tissue blocks (donor blocks), using a TMA instrument (Histopathology Ltd., Hungary) that enabled the cutting of sections of 4 mm in diameter.

### Histopathological analysis

All cases were assessed based on Lauren’s classification and restaged according to the 5th edition of the World Health Organization (WHO) manual for Digestive System Tumors^14^. In addition to TNM (tumor, node, metastasis) stage, the cases were also classified based on the Dukes-MAC-like staging system proposed in 2017 by Gurzu et al*.*^4,15^. Budding index at the tumor front of invasion was assessed for adenocarcinomas according to the International Tumor Budding Consensus: low budding index (b1): 1–4 buds, intermediate (b2): 5–9 buds, and high (b3): ≥ 10 buds^16^. All poorly cohesive (diffuse) carcinomas were classified as b3. Based on lymph node ratio (LNR) value and previous data from literature^17^, cases were classified into three categories: (i) LNR = 0 (no regional lymph node metastases), (ii) LNR = 0.01–0.25 and (iii) LNR ≥ 0.26.

### Immunohistochemical assessment

Immunohistochemical (IHC) examinations were done using antibodies for VSIG1 (rabbit polyclonal HPA036311; Sigma-Aldrich; dilution 1:200), E-cadherin (clone NCH-38; Dako Agilent Technologies, Inc.; dilution 1:50), and β-catenin (clone β-catenin-1; Dako Agilent; dilution 1:150). The TMA sections (5 µm thickness) were deparaffinized and rehydrated, followed by endogenous peroxidase blocking (incubation for 5 min at room temperature with EnVision™ FLEX Peroxidase Blocking solution). Antigen retrieval was performed at high temperature, for 30–40 min, with High pH retrieval solution. This was followed by a 20-min incubation at room temperature with Dako EnVision™ FLEX/HRP detection reagent. EnVision™ FLEX diaminobenzidine (DAB) was used to develop the stains and counterstaining of nuclei was done with Mayer’s hematoxylin.

Since all cases were assessed on TMA blocks, a cut-off value of 15% was used for all the three IHC antibodies, to consider a case as positive. Hepatic tissue served as an external positive control for membrane E-cadherin and β-catenin and for cytoplasmic VSIG1^18^. Normal gastric mucosa was used as a control tissue for membrane VSIG1.

**Figure 1:**
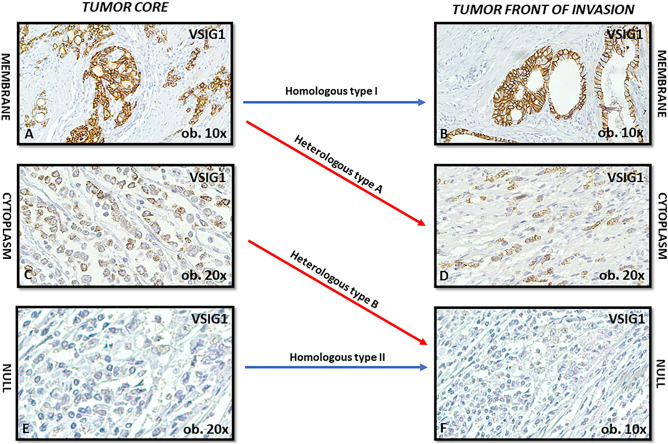
VSIG1 can exhibit three patterns of expression in tumor core versus front of invasion: homologous membrane-membrane (type I), homologous null-null (type II) and heterologous immunostaining (type A: membrane/cytoplasm and type B: cytoplasm in core/null in the invasive front).

The classification of the cases took into account the subcellular pattern of VSIG1 in tumor cells, in the core vs. the invasion front. Based on the subcellular patterns (core vs. front) we grouped the cases into three categories: homologous pattern type I—membrane positivity in both the tumor core and at the front of invasion; homologous pattern type II—null reaction in both core and front (negative cases); and heterologous pattern, with two variants: type A—membrane positivity in the tumor core, with cytoplasmic translocation at the tumor front and type B—cytoplasmic positivity in the tumor core, with loss of VSIG1 (negative) at the front of invasion (Fig. 1).

E-cadherin and β-catenin membrane expression were considered markers of an epithelial phenotype, while cases with membrane-to-nuclear translocation of β-catenin and loss of E-cadherin expression at the tumor front (buds) were considered tumors with EMT, as in our previous studies^19^.

### Statistical analysis

The included cases were statistically assessed using GraphPad Prism software (version 8, software-free version). To determine the association between clinicopathological features and IHC markers, multivariate analysis methods (the Pearson χ^2^), as well as chi-square and Fisher’s exact tests were used, with p values less than 0.05 being considered statistically significant. Cox proportional hazard regressions were applied to estimate the individual hazard ratio (HR) for overall survival (OS), OS rates being estimated using the same software and Kaplan–Meier curves. The median follow-up was 31 months (range 3–59 months) after surgery.

### Institutional review board statement

The Approval of Ethical Committee of Clinical Emergency Hospital of Targu Mures, Romania was obtained for performing the present study.

### Informed consent

Informed consent was taken from every patient included in the prospective database. Part of the study was retrospective.

## Results

### Clinicopathological parameters

Most of the patients included in this study were over 60 years (range 39–86, with a mean age of 65.5 years). The male to female ratio was 3.47:1. It was a statistically significant predominance of location on the distal stomach (p = 0.013). Half of the tumors were differentiated adenocarcinomas with lymph node metastases (pN1–3) at the time of diagnosis (Table 1). 75% of tumors were assessed as type I (intestinal phenotype) according to Lauren’s classification; the other 25% were type II (diffuse/poorly-cohesive) carcinomas (Table 2). The mean LNR was 0.28 ± 0.08. Lymphovascular and perineural invasions were present in 78.9% and 61.7% of cases, respectively (Table 2).

**Table 1:** Clinicopathological features of the 94 gastric cancer cases. Significant values are in bold.

Parameter	Number/% (n = 94)	p value
Median age (standard deviation)	69 years (± 9.14)	–
**Gender**		
Male	73/77.6%	**0.013**
Female	21/22.4%	
**Tumor location**		
Proximal	28/29.79%	**0.038**
Distal	66/70.21%	
**Histologic type and grade of differentiation (G)**		
G1/2 adenocarcinoma	46/48.9%	0.063
G3 adenocarcinoma	25/26.6%	
Poorly cohesive (diffuse) carcinoma	23/24.5%	
**Depth of invasion (pT stage)**		
pT1	10/10.6%	**0.009**
pT2	6/6.4%	
pT3	23/24.5%	
pT4a	42/44.7%	
pT4b	13/13.8	
**Regional lymph node status (pN stage)**		
pN0	26/27.6	**0.033**
pN1–3	68/72.4%	
**Distant metastases (pM stage)**		
M0	77/81.9%	**0.008**
M1	17/18.1%	
**Dukes-MAC-like stage**		
A1 (pT1N0)	9/9.6%	0.056
A2 (pT1N1–3)	1/1.1%	
B1 (pT2N0)	6/6.4%	
B2(pT2N1–3)	–	
C1 (pT3N0)	5/5.3%	
C2 (pT3N1–3)	18/19.2%	
D (pT4N0–3)	55/58.4%

**Table 2:** VSIG1 pattern of expression is statistically significant correlated to regional lymph node status, lymph node ratio and Dukes-MAC-like stage, but not to tumor depth of invasion. At the same time, β-catenin and E-cadherin statuses are significantly different between the three VSIG1 patterns. Significant values are in bold.

	VSIG1 homologous type I (n = 20)	VSIG1 homologous type II (n = 54)	VSIG1 heterologous (type A and B) (n = 20)	p value
**Gender**				
Male	16/80%	39/72.22%	18/90%	0.254
Female	4/20%	15/27.78%	2/10%	
**Age**				
≤ 60	6/30%	8/14.81%	5/25%	0.294
> 60	14/70%	46/85.19%	15/75%	
**Lauren’s histologic subtype**				
Intestinal type				
G2 Adeno-carcinoma	11/55%	27/50%	8/40%	0.356
G3 Adeno-carcinoma	4/20%	17/31.48%	4/20%	
Diffuse/poorly cohesive subtype	5/25%	10/18.52%	8/40%	
**pT stage**				
Early pT (pT1–2)	5/25%	9/16.66%	1/5%	0.219
Advanced pT (pT3–4)	15/75%	45/83.34%	19/95%	
**pN stage**				
N0	8/40%	15/27.78%	1/5%	**0.033**
N1+	12/60%	39/72.22%	19/95%	
**pM stage**				
Mx	19/95%	42/77.78%	16/80%	0.224
M1	1/5%	12/22.22%	4/20%	
**Resection margins**				
R0	17/85%	44/81.48%	14/70%	0.444
R1	3/15%	10/18.52%	6/30%	
**Lymphovascular invasion**				
LV0	6/30%	12/22.22%	2/10%	0.292
LV1	14/70%	42/77.78%	18/90%	
**Perineural invasion**				
n0	10/50%	21/38.88%	5/25%	0.264
n1	10/50%	33/61.12%	15/75%	
**Tumor budding in front of invasion**				
b1	5/25%	10/18.52%	2/10%	0.462
b2	4/20%	19/35.18%	5/25%	
b3	11/55%	25/46.3%	13/65%	
**Dukes-MAC-like stage**				
Early (A–B–C1)	8/40%	10/18.52%	1/5%	**0.020**
Advanced (C2–D)	12/60%	44/81.48%	19/95%	
**Lymph node ratio**				
0.00	9/45%	15/27.78%	1/5%	**0.0022**
0.01–0.25	6/30%	16/29.62%	2/10%	
≥ 0.26	5/25%	23/42.6%	17/85%	
**EMT markers**				
β-catenin expression				
Membrane	19/95%	48/88.9%	11/55%	**0.0007**
Nucleus	1/5%	6/11.1%	9/45%	
E-cadherin expression				
Membrane	18/90%	47/87%	11/55%	**0.004**
Negative	2/10%	7/13%	9/45%	

**Figure 2:**
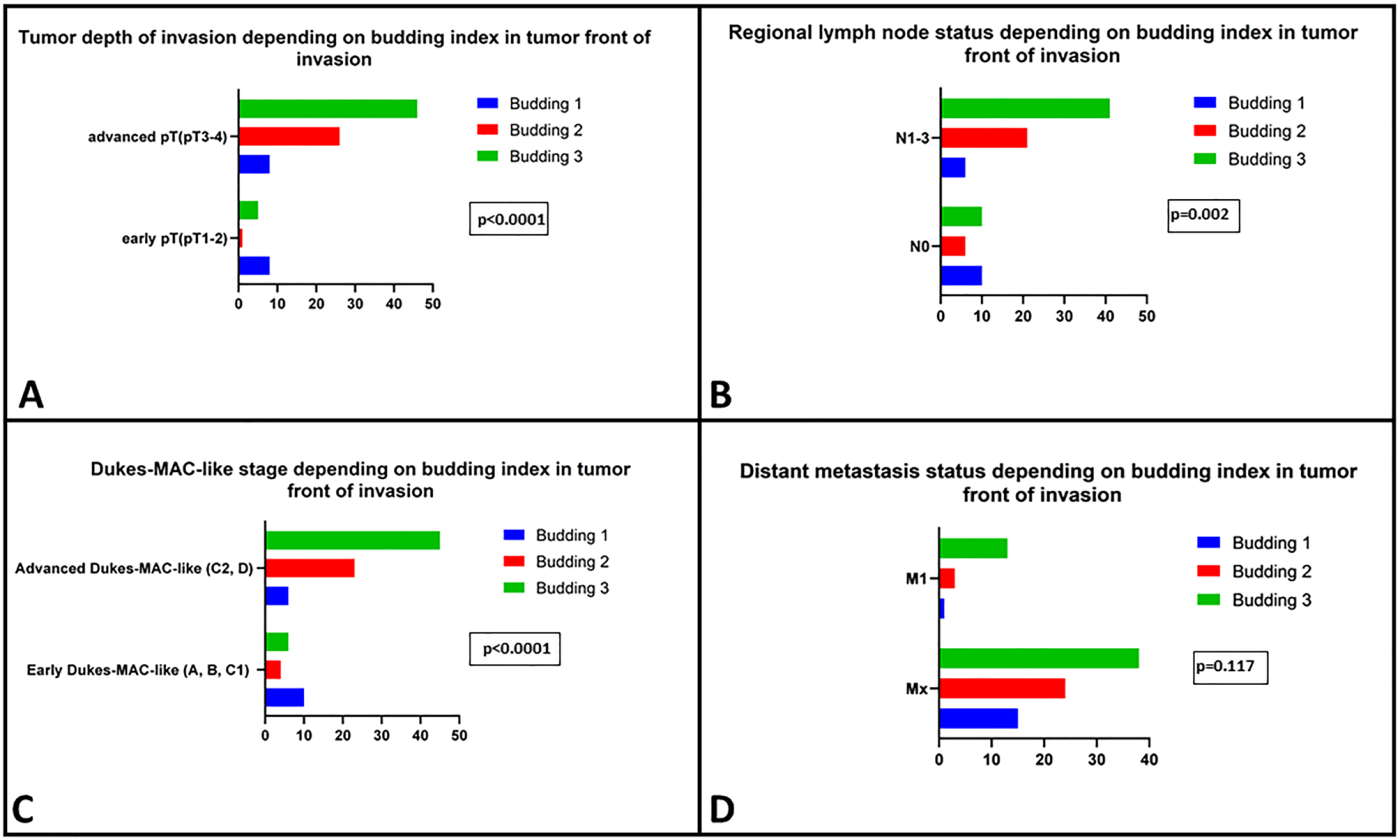
Budding index in the invasive edge of gastric cancer is directly associated with depth of infiltration (**A**), nodal status (**B**) and Dukes-MAC-like stage (**C**) but does not influence the risk for distant metastases (**D**).

Regarding the invasive edge, high-grade budding adenocarcinomas (b3) were mostly de-differentiated (25 G3 and 1 G2). All diffuse carcinomas (n = 23) were also considered as b3. A high budding index at the invasion front was associated with lymphovascular (p = 0.0009) and perineural invasion (p = 0.003) but it was not linked with the risk of distant metastases (p = 0.11). Tumors with advanced pT stage (T3–4), as well as those with advanced Dukes-MAC-like stage (C2–D), were associated with a high budding index at the invasion front. The positive association of the score of b3 confirmed it with the presence of lymph node metastases (Fig. 2).

### VSIG1 subcellular localization

**Figure 3:**
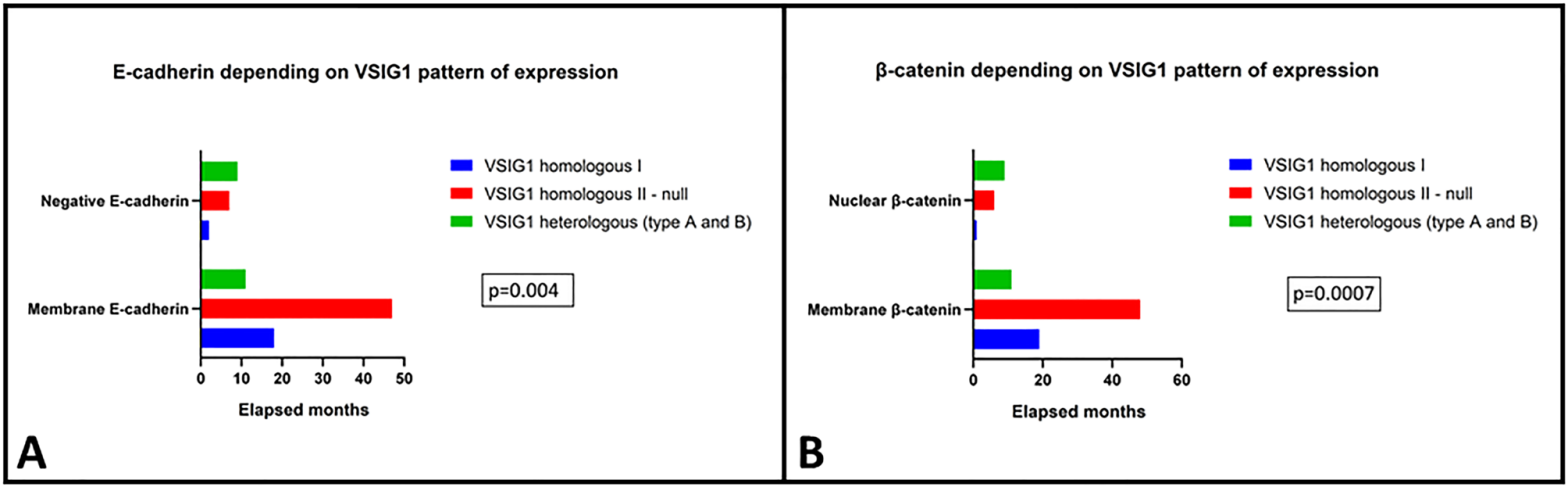
Tumors with homologous patterns of VSIG1 expression are more commonly characterized by double E-cadherin (**A**) and β-catenin membrane positivity (**B**) which reflects an epithelial molecular subtype and absence of epithelial–mesenchymal transition.

**Figure 4:**
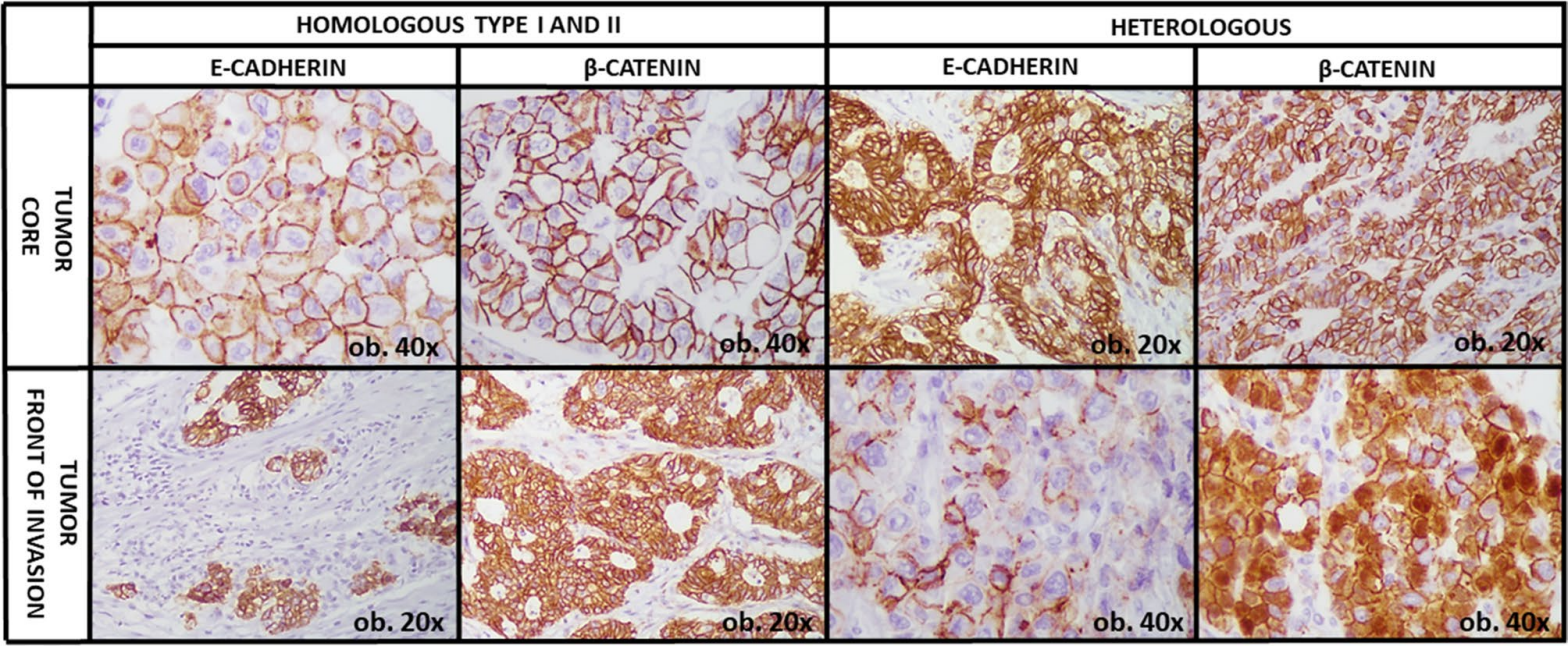
Homologous type I and II patterns of VSIG1 expression exhibit an epithelial phenotype, with synchronous membrane expression for E-cadherin and β-catenin. In contrast, VSIG1-heterologous cases are rather mesenchymal-type, with total or partial loss of E-cadherin and membrane-nuclear translocation of β-catenin.

From all the examined clinicopathological factors, the VSIG1 expression pattern showed a statistically significant correlation with regional lymph node status, LNR, and Dukes-MAC-like stage (Table 2). Of the 94 included cases, only 28 were diagnosed in the proximal part of the stomach: 10 belonged to the heterologous types A and B (r = 1.00) and 18 to the homologous type II group (r = 0.38). Tumor localization was found to slightly influence VSIG1 reactivity (p = 0.04) which was associated with the EMT phenomenon described below in the text (Figs. 3 and 4).

*Homologous type I pattern* was displayed by 20 GCs, mainly occurring in tumors diagnosed in males over 60 years, independent of the histologic subtype or grade of differentiation. This immunophenotype was associated with an early Dukes-MAC-like stage and a lower LNR compared with the other groups. Only one of the 17 cases with distant metastases belonged to this group. VSIG1 membrane positivity in core and invasive edge mostly associated E-cadherin and β-catenin membrane positivity, as indicators of epithelial phenotype (Table 2 and Fig. 3).

*Homologous type II pattern* was displayed by 54 GCs. These were mostly epithelial adenocarcinomas, similar to the homologous type I cases (Fig. 3), but showed a higher tendency for lymph node (high LNR) and distant metastases, compared with the first group, especially for cases with over 5 buds at the invasion front (Table 2).

*Heterologous pattern* was found to be significantly associated with factors of aggressiveness, including advanced stage, high LNR, and risk of distant metastases. Type B was predominant (n = 15); only 5 cases belonged to type A group. Although not statistically significant, this immunophenotype was rather characteristic for poorly cohesive and de-differentiated (G3) adenocarcinomas with over 10 buds at the invasion front (Table 2).

Presence of the EMT in the invasive edge, with total or partial loss of E-cadherin and membrane-nuclear translocation of β-catenin (Fig. 4) reflected a mesenchymal phenotype which was not expressed by the other two patterns (Fig. 3).

### Survival rate

**Figure 5:**
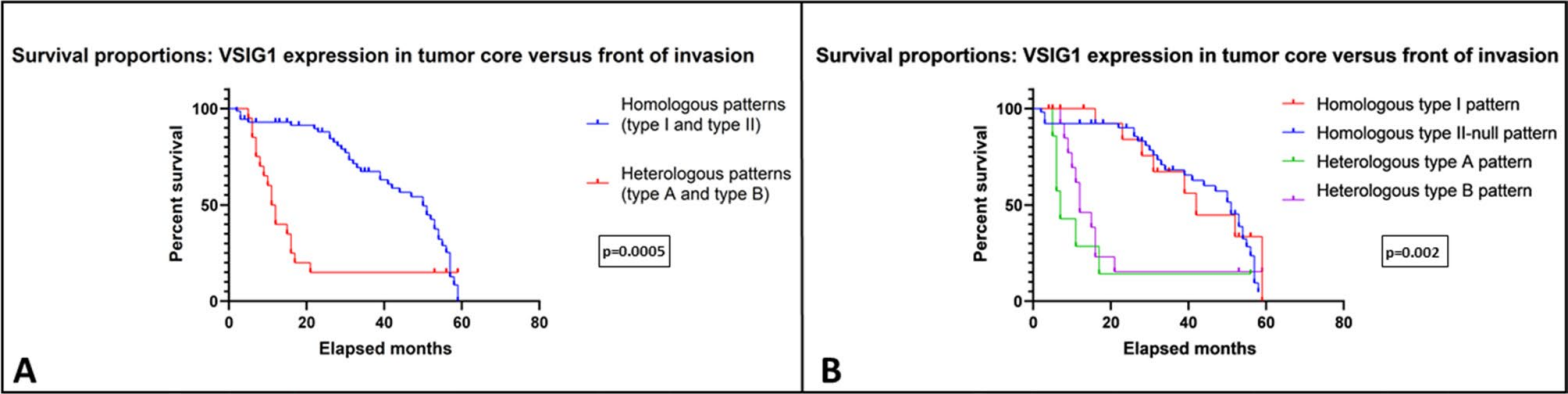
Overall survival rate (OS) is influenced by the subcellular localization of VSIG1. Homologous patterns associate a better OS, compared with the heterologous tumors (**A**). No differences occur between heterologous type A vs. B either between homologous type 1 vs. 2 (**B**).

Kaplan–Meier curves showed that the OS was correlated with presence of lymph node metastases (p = 0.002) and an advanced (C2–D) Dukes-MAC-like stage (p = 0.003). No correlations with tumor location (p = 0.12), pT stage (p = 0.07) or budding index was found (p = 0.49). A shorter OS for patients with heterologous pattern of VSIG1 expression (A and B) was seen, when compared to those with homologous type I and II. The Cox proportional hazard model was used as a regression model to investigate the prognostic value of VSIG1 expression patterns, as follows: VSIG1 homologous type I pattern does not significantly influence the survival, compared to homologous type II pattern (hazard ratio (HR) = 0.96, p = 0.126). On the other hand, both A and B heterologous patterns showed an almost complete overlapping in terms of OS curves, with a HR of 1.09, p = 0.04 and HR of 1.07, p = 0.03 (Fig. 5).

## Discussion

VSIG1 is a relatively newly discovered protein whose role is, to date, incompletely understood. It is a member of the Immunoglobulin superfamily. Like other members of this family, it possesses an extracellular, immunoglobulin-like domain, with a role in intercellular adhesion, and a C-terminal cytoplasmic domain, bounded by a transmembrane region^12^. This subcellular distribution could explain the membrane-to-cytoplasm translocation of VSIG1 revealed in this study in GC.

In normal human tissues, VSIG1 was initially found to be expressed only in normal gastric mucosa and then in testis^18,20,21^. In the 12 published studies in total, from which three were focused on the GC^10,12,13^, it was highlighted that other normal tissues and few epithelial tumors can also display VSIG1 positivity^8–11,18–21^. In normal gastric mucosa and GCs, only membrane expression was previously quantified. Loss of membrane expression was seen to associate a worse evolution^10,12,13^.

Cytoplasmic granular/dot-like VSIG1 immunostaining was firstly proved by our team, during a Romanian-Japan interdisciplinary research project, in normal hepatocytes and a histologic subtype of hepatocellular carcinoma (HCC) which was called by the researchers as “gastric-type HCC”^18^. As this is the first report about membrane to cytoplasmic translocation of VSIG1 in GC, it is difficult to find a reliable explanation for the unusual translocation.

**Figure 6:**
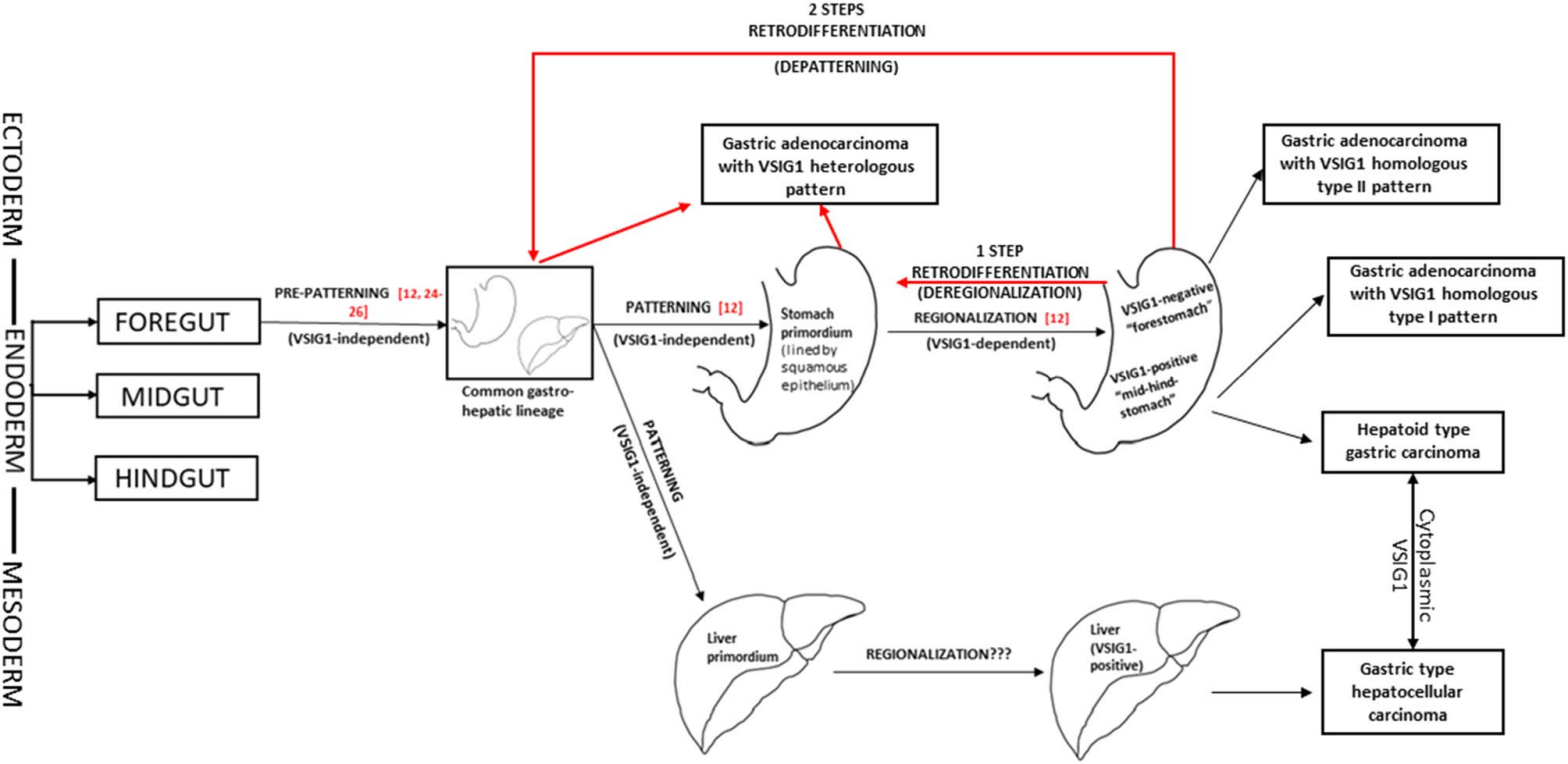
VSIG1-related theory of gastric tumorigenesis—designed based on own data and information from papers of Oidovsambuu et al.^12^ and Gurzu et al.^18^.

Because the gastric-type HCC and hepatoid-type of GC seems to share a common origin, in the endodermal rest of the foregut, as we have already hypothesized^18,22,23^, it is worth considering whether the presence of VSIG1 cytoplasmic immunostaining in HCC cells is a sign of gastric differentiation, or the reverse, that cytoplasmic pattern of VSIG1 expression in GC cells is a sign of hepatoid differentiation. Nevertheless, cytoplasmic immunostaining should be interpreted as an indicator of retro-differentiation towards a common “hepato-gastric” embryologic lineage (Fig. 6). This mechanism might explain why the heterologous GCs were associated with the parameters of aggressiveness and had a poor OS compared to those with homologous patterns.

This theory can help understanding occurrence of the cytoplasmic stain in some heterologous cases. It is still difficult to understand why some VSIG1-negative cases (homologous type II) showed a similar behaviour with the cases with diffuse membrane immunoreactivity (homologous type I).

On the one hand, the embryologic endodermal development can be, at least mechanistically, divided into two different processes: (i) the pre-patterning of endoderm into foregut, midgut, and hindgut, followed by (ii) the regionalization of those three embryologic structures by the end of gastrulation. The pre-patterning process was proven to be regulated by several transcription factors^24–26^. VSIG1, on the other hand, act as a regulator of the (ii) regionalization process, being indispensable for the differentiation of glandular gastric epithelium. Due the similarities between VSIG1 patterns of cytoplasmic expression in gastric- and hepatoid type carcinomas, it is, in our opinion, highly probable to exist a molecular linkage from developmental perspective between those two tumors. The same stepwise mechanisms might be involved in gastric and hepatic carcinogenesis as those above-mentioned, but in a reverse manner. So, in light of those, our theory is centered on two different, but continuous processes of retrodifferentiation: a one-step retrodifferentiation towards stomach/hepatic primordiums—*deregionalization* and a two-step retrodifferentiation—*depatterning* towards a common gastro-hepatic primordium (Fig. 6).

The histologic transition between “forestomach” and “mid-hindstomach” is overlapped by the transition between VSIG1-negative squamous epithelium, covering the initial segment of stomach primordium, and VSIG1-positive gastric-type glandular epithelium, present on the remnant gastric primordium^12^. Although this remains to be demonstrated, the theory of regionalization-dependent tumors might explain why some of VSIG1 null GCs (homologous type II pattern) still exhibit an OS rate similar to that of homologous type I cases (membrane positivity) but loss of membrane positivity in the invasion front (heterologous type A) associate aggressive behaviour.

According to this embryologic theory, homologous type II cases with good OS should be located preferentially in the proximal segment of the stomach, a hypothesis that was partially confirmed in our study. These tumors did not lose the positivity (as a sign of EMT) but they were rather developed from rests of VSIG1 negative pre-patterning endodermis. When they derive from the embryological rests of VSIG1 positive regionalized endodermis, they keep the membrane positivity (homologous type A) and associate slowly metastatic behaviour. In these cases, if the EMT is activated (heterologous VSIG1), it induces loss of cohesivity with a high-grade budding index and increased invasiveness. We were not able to find any data in the literature to support our hypothesis, but this theory deserves further attention, particularly in larger cohorts. In carcinogenesis, however, members of tight junctions family, of which the adhesion protein VSIG1 is a part, have gained much attention in recent decades^13,27^.

Another interesting result of this paper was the correlation between cytoplasmic expression of VSIG1, nuclear translocation of β-catenin and loss of E-cadherin expression. Considering that E-cadherin loss and membrane-to-nuclear β-catenin translocation are indicators of EMT^19,28,29^, it can be stipulated that VSIG1 cytoplasmic positivity (heterologous type A and B) could be also considered surrogate markers for EMT. Having similar patterns of staining to β-catenin and E-cadherin, it is probable that VSIG1 plays a role in EMT by modulating *Wnt* pathway in a similar fashion. Yet to be demonstrated, with the confirmation of this hypothesis, VSIG1 would become an even more intriguing molecule, having two opposite roles: in embryogenesis, it might act as a glandular-epithelial differentiation marker, but in carcinogenesis, its role is diverted, becoming an EMT activator, a marker of mesenchymal phenotype. It was already demonstrated that acquisition of epithelial cell plasticity is intimately related to multiple changes in *Wnt* pathway^30,31^. In canonical *Wnt* pathway, the key regulator is *β catenin,* of which accumulation in the cytoplasm and then nuclear translocation, leads to the activation of specific transcription factor of TCF/LEF family^32^. As this is, to the best of our knowledge, the first study to raise the possibility of VSIG1 involvement in tumor cell plasticity, specifically in EMT and *Wnt* pathway in a similar fashion to β catenin, further research is needed to establish whether the same transcriptional patterns are used by VSIG1, or the molecular machinery is different, at least in part.

Regarding the general clinical-pathological features, our study demonstrated similar results to those published in literature: in our cohort, factors of aggressiveness were found to be a high lymph node ratio, with a propensity towards poorly differentiated adenocarcinomas or diffuse carcinomas, as well as an advanced Dukes-MAC-like stage^15^. Also, as expected, the overall survival was negatively influenced by the presence of lymph node metastasis/higher lymph node ratio, and distant metastases.

In conclusion, when located in the membrane of tumor cells, VSIG1 acts as an adhesion membrane protein, as it was previously highlighted. Although the mechanism of membrane-cytoplasmic translocation of VSIG1 is not known, it is probably induced by VSIG1-mediated activation of canonical Wnt/β-catenin signaling pathway. The hypothesis needs to be validated by further experimental studies.

Limitations of the study consist in the small cohort but, as the senior author is an experienced gastrointestinal pathologist, she can certify the results. We started this VSIG1 observation in 2014 but did not publish the result due to the scarce data from literature which did not allow external validation. Other limitation is lack of statistical correlation between VSIG1 expression and molecular classification of GC. This was not done because only 6 of the 94 cases showed microsatellite instability and Epstein–Barr-induced carcinomas are quite rare in our geographic area^33^.

## Data Availability

The database details are available at the corresponding authors upon request.
